# The abdominal aortic aneurysm-related disease model based on machine learning predicts immunity and m1A/m5C/m6A/m7G epigenetic regulation

**DOI:** 10.3389/fgene.2023.1131957

**Published:** 2023-02-23

**Authors:** Yu Tian, Shengjie Fu, Nan Zhang, Hao Zhang, Lei Li

**Affiliations:** ^1^ Department of Vascular Surgery, The Second Hospital of Dalian Medical University, Dalian, China; ^2^ Department of Thyroid and Breast Surgery, Tongji Hospital, Tongji Medical College of Huazhong University of Science and Technology, Wuhan, China; ^3^ Department of Neurosurgery, The Second Affiliated Hospital, Chongqing Medical University, Chongqing, China

**Keywords:** aortic aneurysm, disease model, m1A/m5C/m6A/m7G, immunity, machine learning

## Abstract

**Introduction:** Abdominal aortic aneurysms (AAA) are among the most lethal non-cancerous diseases. A comprehensive analysis of the AAA-related disease model has yet to be conducted.

**Methods:** Weighted correlation network analysis (WGCNA) was performed for the AAA-related genes. Machine learning random forest and LASSO regression analysis were performed to develop the AAA-related score. Immune characteristics and epigenetic characteristics of the AAA-related score were explored.

**Results:** Our study developed a reliable AAA-related disease model for predicting immunity and m1A/m5C/m6A/m7G epigenetic regulation.

**Discussion:** The pathogenic roles of four model genes, UBE2K, TMEM230, VAMP7, and PUM2, in AAA, need further validation by *in vitro* and *in vivo* experiments.

## Introduction

The heart pumps arterial blood through the aorta, then into its branches to supply the body in the abdominal section called the abdominal aorta. An abdominal aortic aneurysm is diagnosed when the diameter of the abdominal aorta enlarges by more than 50% of its diameter for various reasons (Chaikof et al., 2018). It is commonly understood that the abdominal aorta, which was originally straight, has an abnormal expansion, like blowing up a balloon. When the development reaches a certain extent, it may burst suddenly. Therefore, an abdominal aortic aneurysm is sometimes compared to a “bomb with no timing” (Sakalihasan et al., 2005).

Most patients with abdominal aortic aneurysms are asymptomatic. Occasionally, patients find a “pulsing lump” on their stomach by accident or during a visit for another medical condition. If an abdominal aortic aneurysm suddenly causes severe abdominal pain, it is often a sign that it has ruptured or has ruptured. In addition, enlarged aneurysms can compress other organs in the abdominal cavity, such as intestinal compression, causing nausea, vomiting, abdominal distension, and discomfort. Compression of the ureter can cause hydronephrosis and so on.

Abdominal aortic aneurysms (AAA) cause three main harms: 1) The enlarged abdominal aorta compresses the surrounding vital organs and tissues, affecting their physiological functions. 2) Thrombus is easy to form in the lumen, which blocks the lower limb blood vessels after the thrombus falls off, leading to acute ischemic necrosis of the limb, just like the sudden water cut or power cut in daily life. 3) Under the continuous impact of blood flow, the abdominal aorta gradually enlarges, and when it exceeds the maximum tolerance limit, the aneurysm will burst and cause sudden death (Sakalihasan et al., 2005). Although aneurysms and solid tumors are entirely different concepts, the risk of death is more significant once ruptured than any solid tumor (Wanhainen et al., 2019).

Vascular inflammation is the first occurrence of AAA (Marquez-Sanchez and Koltsova, 2022). In the early stages of AAA, immune cells infiltrate and aggregate in the blood vessels, leading to inflammatory responses in the vessel walls. Inflammatory cells stimulate smooth muscle cells to secrete matrix metalloproteinase, which degrades elastin and collagen, reduces the stability of the artery wall, and induces apoptosis of vascular smooth muscle cells, thus playing an essential role in the occurrence and development of AAA (Marquez-Sanchez and Koltsova, 2022). Further understanding the mechanism of regulating immune cell activation in AAA will provide important targets for therapeutic intervention.

As an important research direction of post-transcriptional gene regulation, N1 methyladenosine (m1A), 5-methylcytosine (m5C), N6-methyladenosine (m6A), and 7-methylguanosine (m7G) modified RNA is widely found in eukaryotic cells and plays a vital role in a variety of biological processes (Wu et al., 2021). They also function as a novel mechanism in cardiovascular diseases, including heart failure, coronary heart disease, and hypertension (He et al., 2019b; Wu et al., 2021). However, the specific roles of m1A/m5C/m6A/m7G regulation in AAA have not been fully elucidated.

In this regard, we developed an AAA-related model and provided new mechanism insights for immunity and m1A/m5C/m6A/m7G regulation in AAA using large-scale bioinformatics analysis.

## Methods

### Data collection

GSE98278 (48 AAA samples) and GSE47472 (14 AAA samples and eight normal samples) from the Gene Expression Omnibus (GEO) database were collected and used for the follow-up studies. GSE98278 and GSE47472 were RMA normalized and conformed to a normal distribution.

### WGCNA for the AAA-related genes

Weighted correlation network analysis (WGCNA) was performed on the top 5,000 genes from GSE47472 using the R package WGCNA. Gene significance was used to quantify the relationship between specific genes and macrophage densities, while module membership was used to illustrate the relationship between module eigengenes and gene expression profiles. To guarantee a scale-free topology network and create a TOM matrix, a power of *β* = 16 was automatically generated by the pickSoftThreshold function, and a scale-free R2 = 0.86 automatically derived by the softConectivity function were used as soft-threshold parameters. Using the plotEigengeneNetworks function, the module dendrogram showing the link between the eigengenes and the macrophages was plotted after the module eigengenes had been recalculated. Enrichment analysis for Gene Ontology-Biological Process (GO-BP), Gene Ontology-Cell Component (GO-CC), Gene Ontology-Molecular Function (GO-MF), and Kyoto Encyclopedia of Genes and Genomes (KEGG) pathways related to the genes in the turquoise module was performed in GSE47472 by the R package clusterProfiler.

### Identification of the AAA-related genes

The R package limma identified the differentially expressed genes (DEGs) between AAA and normal samples. The R package pheatmap was used to visualize the DEGs between AAA and normal samples. The R package venn was used to visualize the intersected genes between the limma-based DEGs and the WGCNA-based genes in GSE47472. The R package Pi performed the GSEA for GO pathways related to the AAA-related genes in GSE47472.

### Identification of the AAA-related clusters

The R package ConsensusClusterPlus was used to identify the AAA-related clusters based on the AAA-related genes in GSE98278 using the kmdist method. The R package pheatmap was used to visualize the AAA-related genes in the AAA-related clusters in GSE98278.

### Development of the AAA-related score

The R package limma identified the DEGs between the AAA-related clusters in GSE98278. Random forest (machine learning) was performed for dimension reduction of the limma-based DEGs between the AAA-related clusters in GSE98278. LASSO regression analysis (machine learning) was performed to develop the AAA-related score, which lambda.min = 0.0063. The AAA-related score was calculated as the sum of gene*coefficient. The R package pROC was used to generate the ROC curves of four model genes in predicting the AAA-related clusters in GSE98278.

### Immune characteristics of AAA-related score

The R package pheatmap was used to visualize the association between AAA-related score and CIBERSORT-based immune cells in GSE98278. The R package Pi performed the GSEA for GO pathways related to AAA-related score in GSE98278. m1A-related genes, m5C-related genes, m6A-related genes, and m7G-related genes were collected from the previous study (Li et al., 2022; Shao et al., 2022).

### Induction of AAA mice model

Alzet osmotic minipumps (Model 2004; ALZA Scientific Products, Mountain View, California, United States) were implanted into APOE−/− mice (C57BL/6J) at 8 weeks of age. Pumps were filled either with saline vehicle or solutions of Ang II (Sigma Chemical Co., St. Louis, Missouri, United States) that delivered (subcutaneously) 1,000 ng/min/kg of Ang II for 28 days. Pumps were placed into the subcutaneous space of mice through a small incision in the back of the neck. All procedures involving animals were approved by the Animal Care and Use Committee at Dalian Medical University.

### RT-qPCR assay for validation of four model genes

Six AAA and six normal samples were collected from our mice model. Total RNA was extracted by Trizol. The absorbance was measured at 260 and 280 nm, and the concentration and purity were calculated. The RT-qPCR assay was performed with SYBR method. All the primers were designed with primer 5.0. H-actin (https://www.ncbi.nlm.nih.gov/gene/60; F ACC​CTG​AAG​TAC​CCC​ATC​GAG R AGC​ACA​GCC​TGG​ATA​GCA​AC; Product length 224bp). H-UBE2K (https://www.ncbi.nlm.nih.gov/gene/3093; F AGC​GAG​GAG​ACG​AGC​AAA​AA R ACA​AAT​AGC​CCC​TGT​GAC​GG; Product length 222bp). H-TMEM230 (https://www.ncbi.nlm.nih.gov/gene/29058; F GCT​GTC​AGG​CTA​CAT​CAG​CA R ACC​ACG​GTA​GCC​TTT​GGA​TG; Product length 230bp) H-VAMP7 (https://www.ncbi.nlm.nih.gov/gene/6845; F ACT​TCC​TGG​AGG​ATT​TTG​AAC​G R TGT​CTG​TGC​TCT​TGA​ACC​GT; Product length 95bp). H-PUM2 (https://www.ncbi.nlm.nih.gov/gene/23369; F GGA​ATG​GGA​GAG​ACC​ATT​CAA R CTT​TCT​GAT​CGC​GGA​GAC​AGT; Product length 112bp).

### Statistical analyses

The Wilcoxon or Kruskal–Wallis test was used to compare non-parametric variables, while the *t*-test or one-way ANOVA was used to compare parametric variables. Spearman’s correlation analysis was used to calculate correlation coefficients. All statistical analyses were two-sided, and *p* < 0.05 was considered statistically significant.

## Results

### WGCNA for the AAA-related genes

**FIGURE 1:**
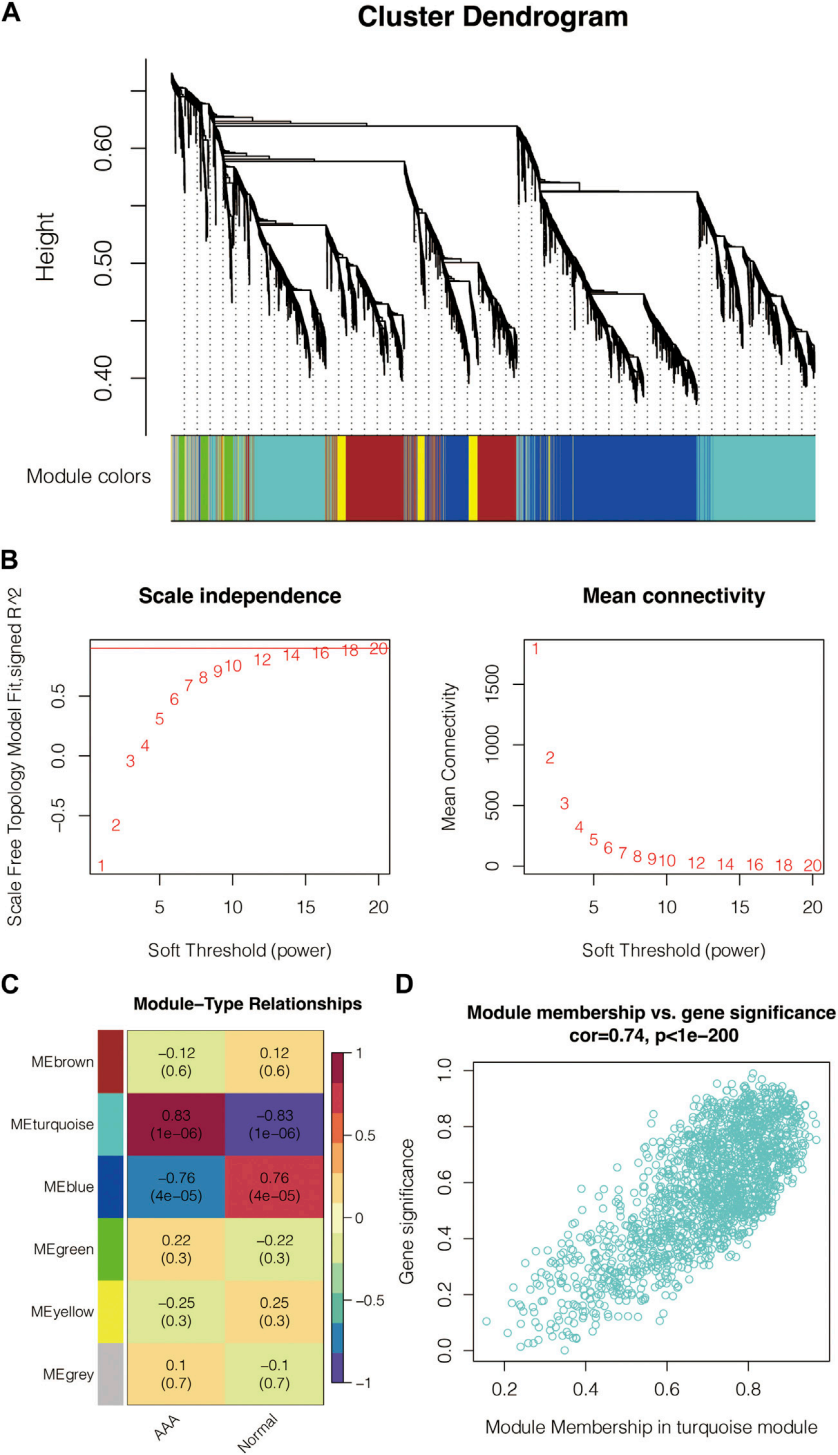
WGCNA for the AAA-related genes. **(A)** Cluster dendrogram of the input genes in GSE47472. **(B)** Scale independence and mean connectivity of the scale-free topology model in GSE47472. **(C)** Module-type relationships in AAA and normal samples in GSE47472. **(D)** Module membership and gene significance in the turquoise module in GSE47472.

**FIGURE 2:**
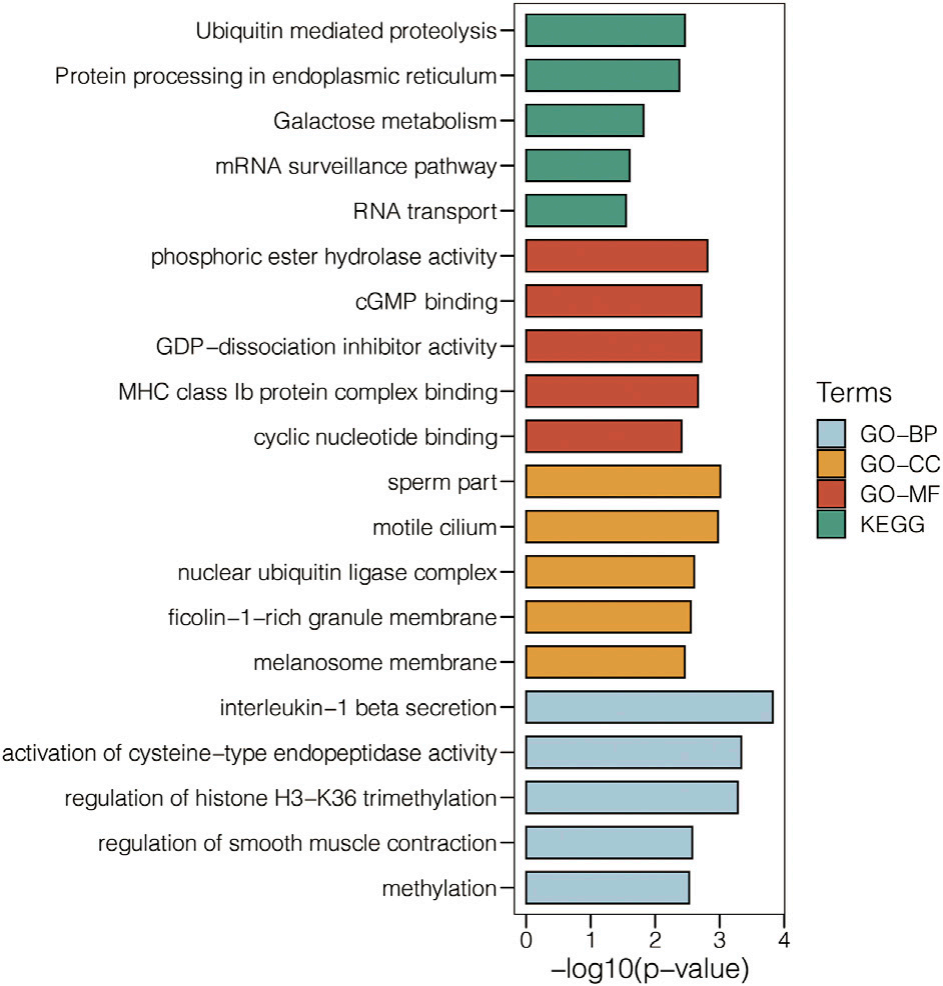
Enrichment analysis for GO-BP, GO-CC, GO-MF, and KEGG pathways related to the genes in the turquoise module in GSE47472.

5,000 genes were used as the input for generating the cluster dendrogram in GSE47472 (Figure 1A). The scale-free topology model’s scale independence and mean connectivity in GSE47472 are calculated (Figure 1B). Module-type relationships in AAA and normal samples in GSE47472 are shown in Figure 1C, in which the turquoise module showed the highest positive correlation with AAA and the highest negative correlation with normal. Module membership and gene significance in the turquoise module in GSE47472 are shown in Figure 1D, in which the correlation coefficient reached 0.74. The module genes in the turquoise module were extracted for enrichment analysis for GO-BP, GO-CC, GO-MF, and KEGG pathways (Figure 2). Notably, Ubiquitin mediated proteolysis, phosphoric ester hydrolase activity, nuclear ubiquitin ligase complex, and interleukin-1 beta secretion were highly enriched.

### Identification of the AAA-related genes

**FIGURE 3:**
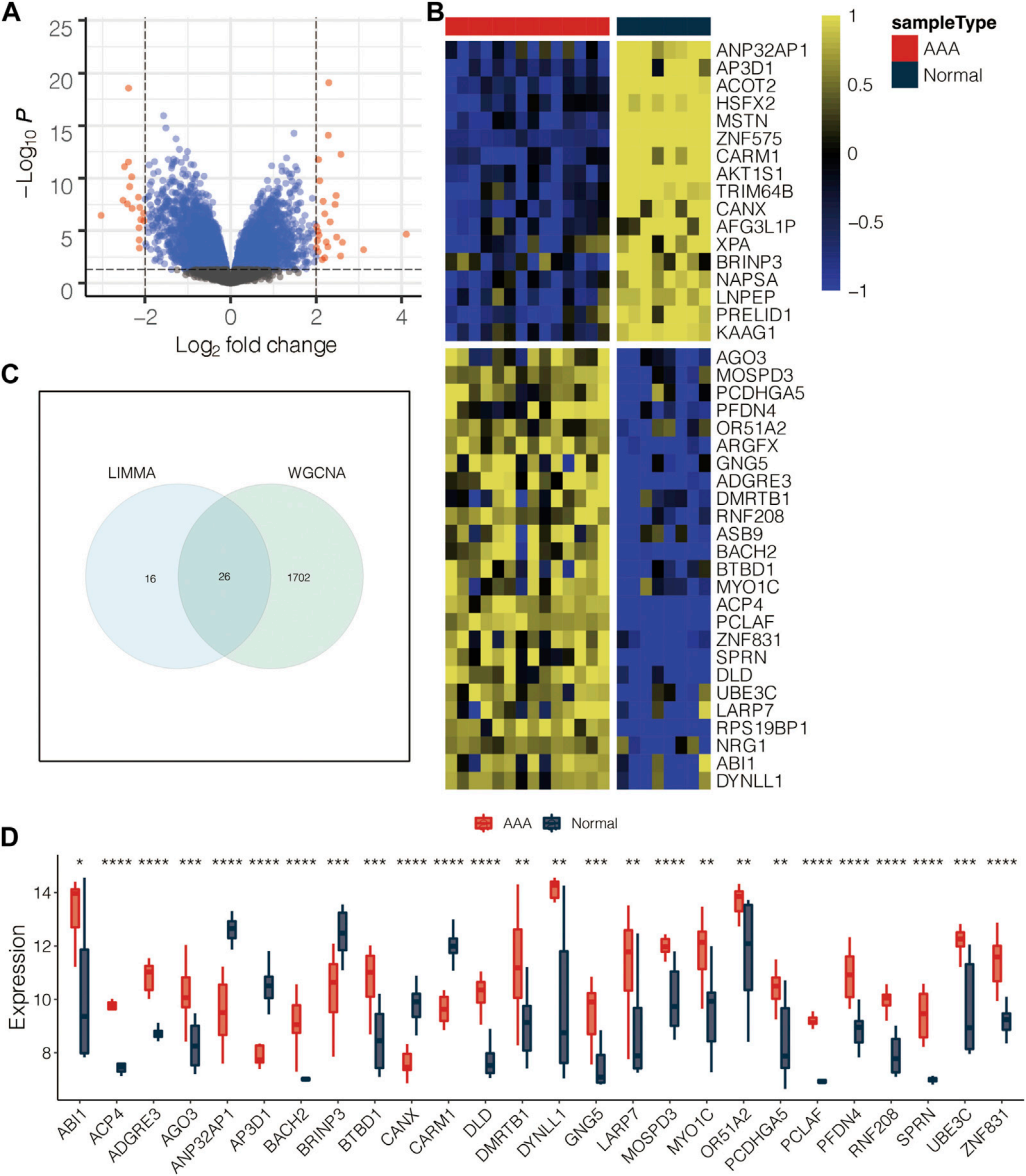
Identification of the AAA-related genes. **(A)** Volcano plot for the limma-based DEGs between AAA and normal samples in GSE47472. **(B)** Heatmap for the DEGs between AAA and normal samples in GSE47472. **(C)** Venn plot for the intersected genes between the limma-based DEGs and the WGCNA-based genes in GSE47472. **(D)** Box plot for the intersected genes between the limma-based DEGs and the WGCNA-based genes in GSE47472.

**FIGURE 4:**
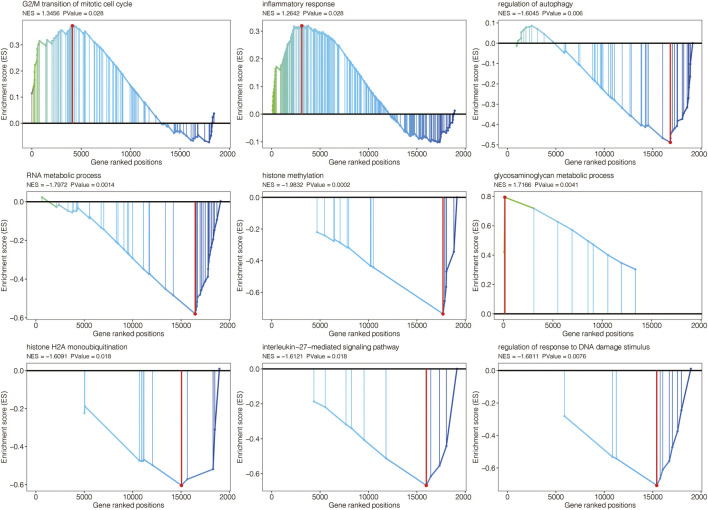
GSEA for GO pathways related to the AAA-related genes in GSE47472.

The volcano plot displayed the limma-based DEGs between AAA and normal samples in GSE47472 (Figure 3A). Heatmap further showed the DEGs between AAA and normal samples in GSE47472 (Figure 3B). Venn plot displayed the 26 intersected AAA-related genes between the limma-based DEGs and the WGCNA-based genes in GSE47472 (Figure 3C). The expression pattern of the 26 intersected AAA-related genes in AAA and normal samples in GSE47472 revealed that the expression of most of the genes was significantly higher in AAA (Figure 3D). GSEA for GO pathways related to the AAA-related genes was performed in GSE47472 (Figure 4). Notably, the G2/M transition of the mitotic cell cycle and inflammatory response were positively enriched. In contrast, regulation of autophagy, RNA metabolic process, histone methylation, glycosaminoglycan metabolic process, histone H2A monoubiquitination, interleukin-27-mediated signaling pathway, and regulation of response to DNA damage stimulus were negatively enriched.

### Identification of the AAA-related clusters

**FIGURE 5:**
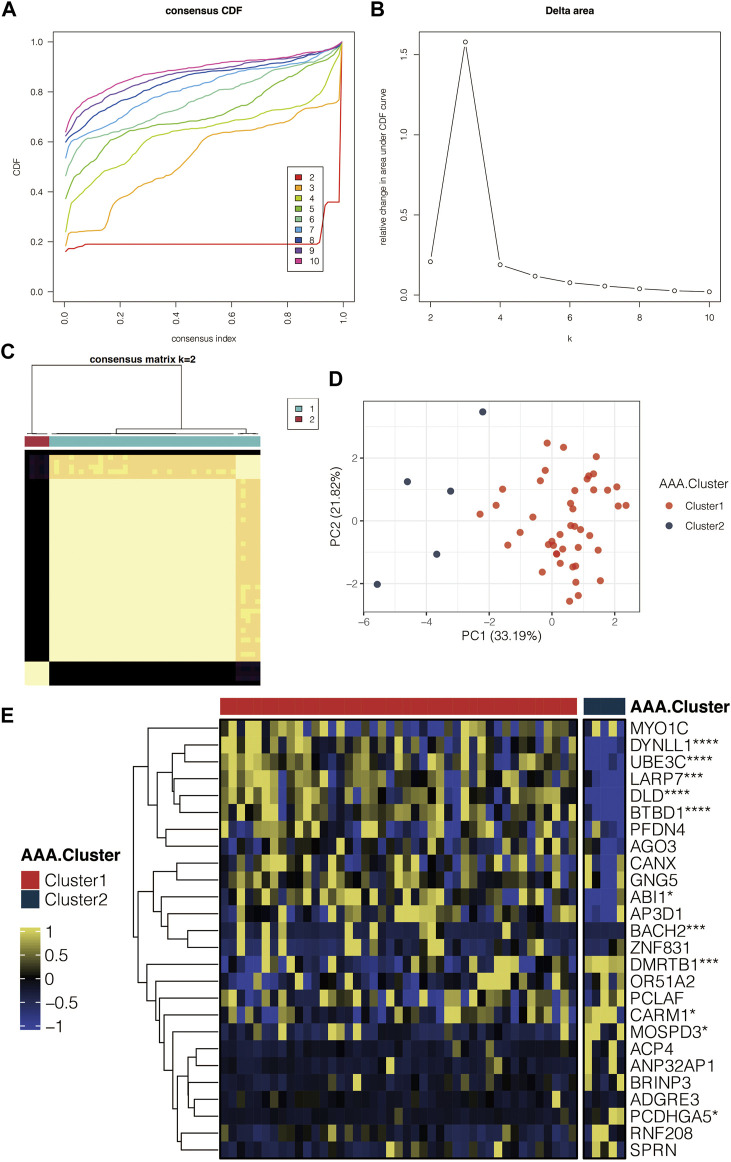
Identification of the AAA-related clusters. **(A)** Consensus CDF curves in GSE98278. **(B)** Delta area in GSE98278. **(C)** Consensus matrix with k = 2 in GSE98278. **(D)** PCA of the AAA-related clusters in GSE98278. **(E)** Heatmap for the AAA-related genes in the AAA-related clusters in GSE98278.

A consensus cluster of AAA samples in GSE98278 was performed based on the AAA-related genes. Consensus CDF curves in GSE98278 revealed the most smooth curve with the k = 2 Figure 5A. The delta area in GSE98278 reflected the relative changes in the area under the CDF curves (Figure 5B). The clustering results were believed to be the most robust with k = 2. The consensus matrix with k = 2 in GSE98278 showed consistency clustering among samples (Figure 5C). The PCA of the AAA-related clusters in GSE98278 revealed that the samples in the two clusters were separated (Figure 5D). Heatmap further displayed the AAA-related genes in the AAA-related clusters in GSE98278 (Figure 5E).

### Development of the AAA-related score

**FIGURE 6:**
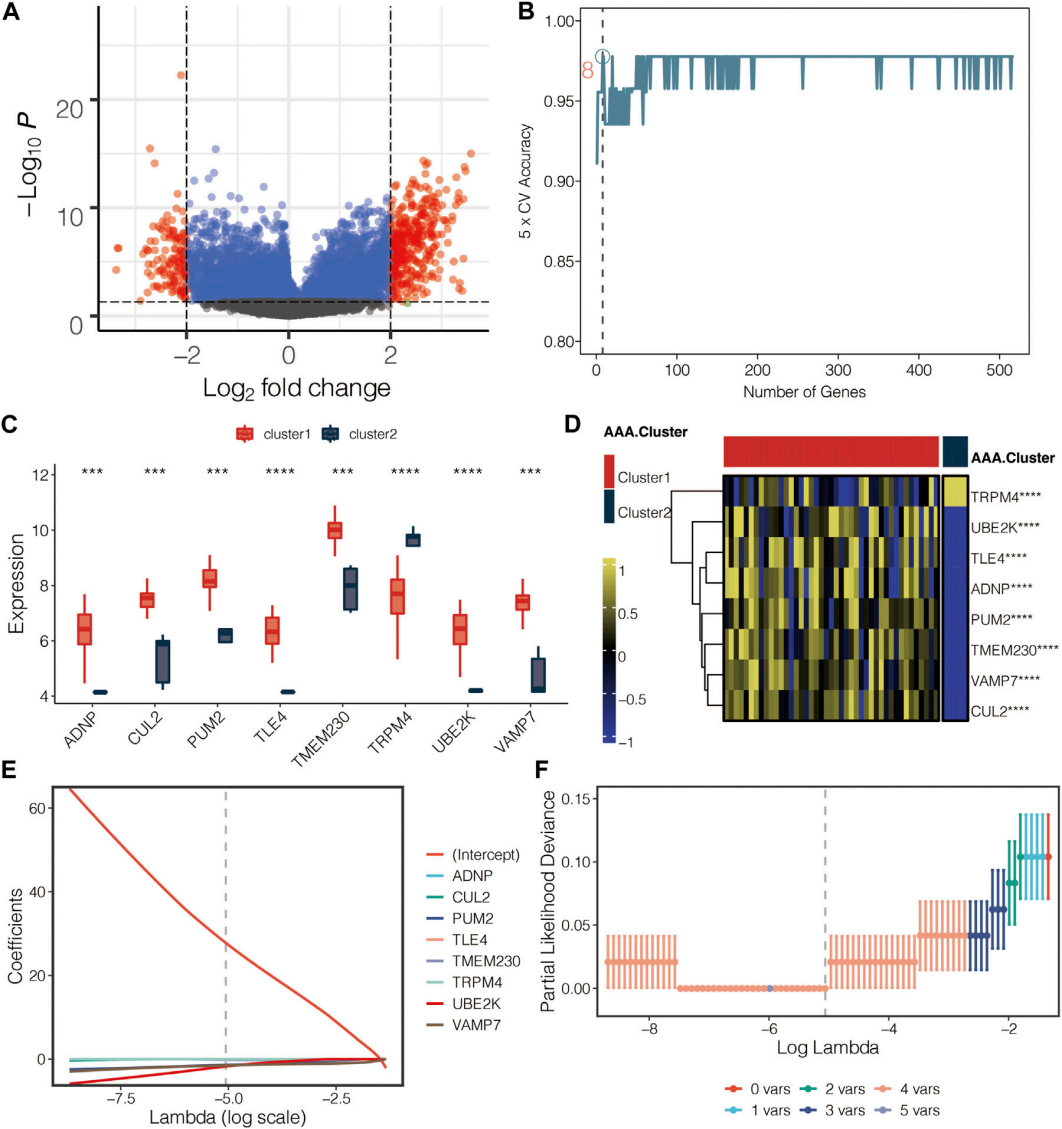
Development of the AAA-related score. **(A)** Volcano plot for the limma-based DEGs between the AAA-related clusters in GSE98278. **(B)** Random forest for dimension reduction of the limma-based DEGs between the AAA-related clusters in GSE98278. **(C)** Box plot for the eight limma-based DEGs between the AAA-related clusters in GSE98278. **(D)** Heatmap for the eight limma-based DEGs between the AAA-related clusters in GSE98278. **(E)** Coefficients in LASSO regression analysis in GSE98278. **(F)** Partial likelihood deviance in LASSO regression analysis in GSE98278.

**FIGURE 7:**
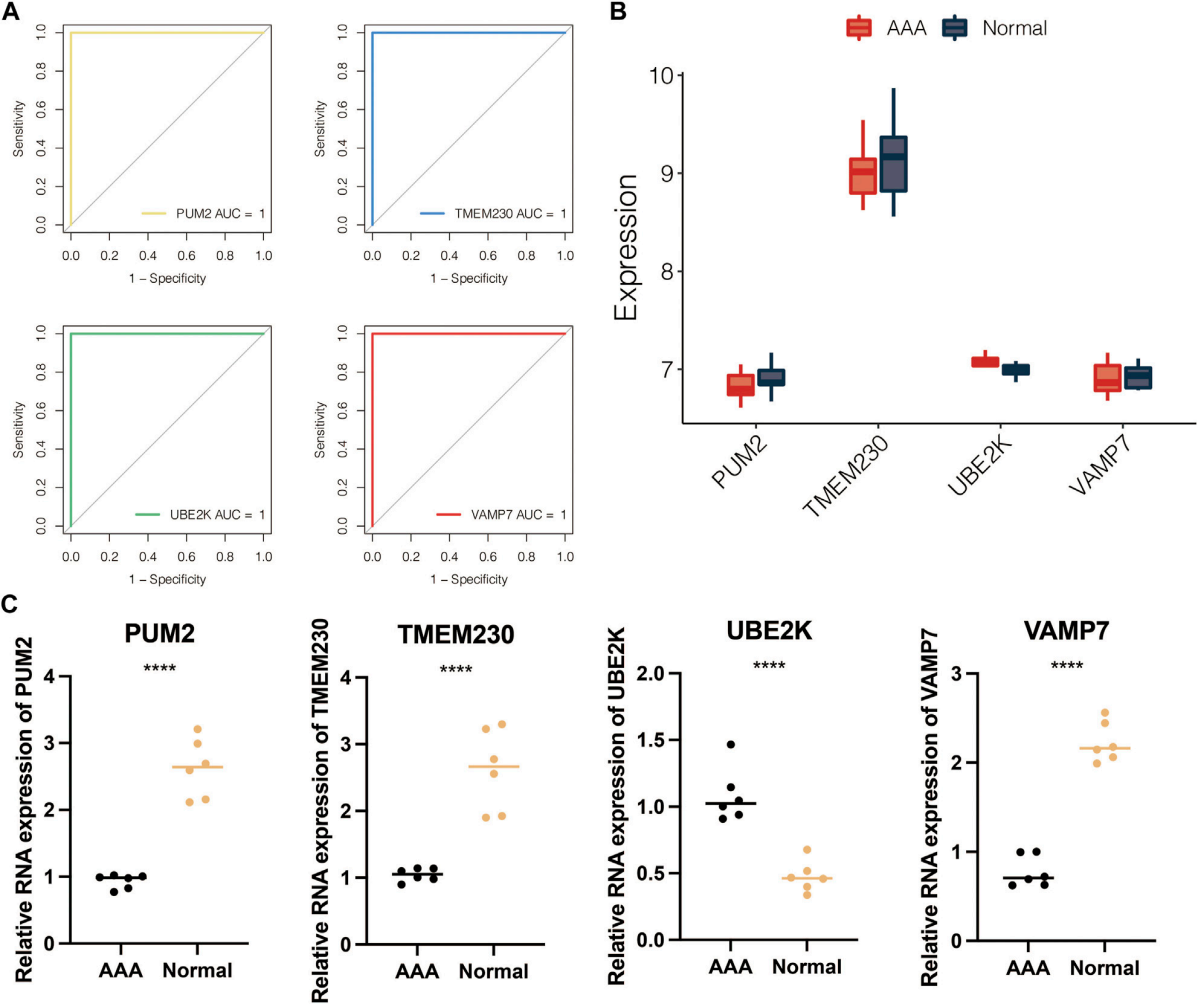
Validation of four model genes. **(A)** ROC curves of four model genes in predicting the AAA-related clusters in GSE98278. **(B)** The expression of four model genes in AAA and normal samples in GSE47472. **(C)** The expression of four model genes in AAA and normal samples by qRT-PCR assay.

The volcano plot displayed the limma-based DEGs between the AAA-related clusters in GSE98278 (Figure 6A). Random forest was performed for dimension reduction of the limma-based DEGs between the AAA-related clusters in GSE98278 (Figure 6B), which came to eight limma-based DEGs between the AAA-related clusters in GSE98278 (Figure 6C). Heatmap further displayed the eight limma-based DEGs between the AAA-related clusters in GSE98278 (Figure 6D). Coefficients in LASSO regression analysis in GSE98278 are shown in Figure 6E. Partial likelihood deviance in LASSO regression analysis in GSE98278 is shown in Figure 6F. The AAA-related score was calculated as follows: −1.7542*UBE2K + −0.1469*TMEM230 + −1.5033*VAMP7 + −1.3816*PUM2 + 27.7793832. The ROC curves of four model genes (UBE2K, TMEM230, VAMP7, and PUM2) predict the AAA-related clusters in GSE98278 (Figure 7A). The expression of PUM2, TMEM230, and VAMP7 was relatively lower in AAA compared with normal samples in GSE47472 (Figure 7B). The expression of four model genes in AAA and normal samples was further validated by qRT-PCR assay. In accordance with the previous finding, the expression of PUM2, TMEM230, and VAMP7 was relatively lower in AAA compared with normal samples in our mice model (Figure 7C).

### Immune characteristics of AAA-related score

**FIGURE 8:**
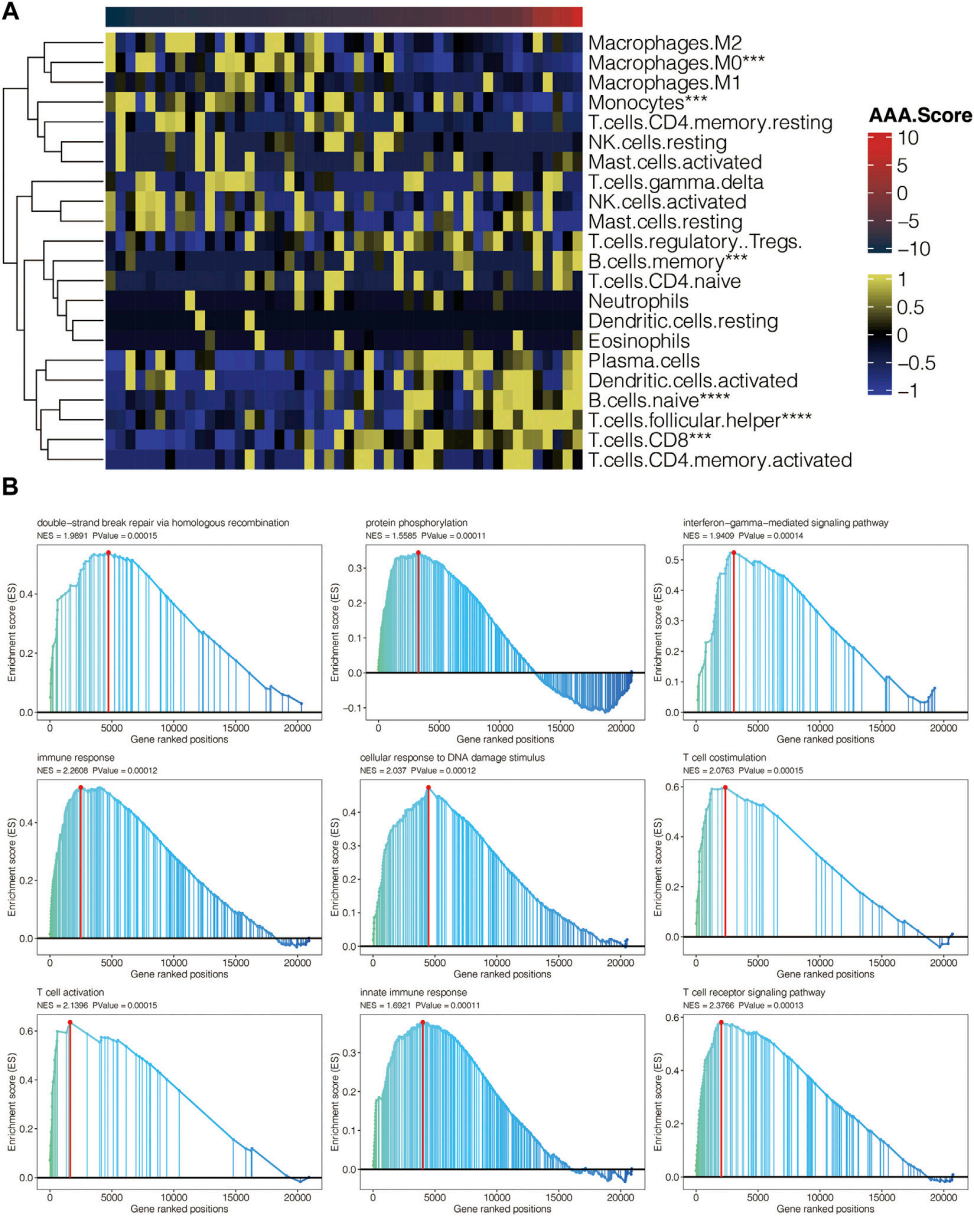
Immune characteristics of AAA-related score. **(A)** Heatmap for the association between AAA-related score and CIBERSORT-based immune cells in GSE98278. **(B)** GSEA for GO pathways related to AAA-related score in GSE98278.

Heatmap displayed the association between AAA-related score and 22 CIBERSORT-based immune cells in GSE98278 (Figure 8A), in which AAA-related score positively correlated with plasma cells, activated dendritic cells, memory B cells, naïve B cells, activated CD4 memory T cells, and CD8 T cells while negatively correlated with macrophage M0, macrophage M1, macrophage M2, and monocytes. GSEA was performed for GO pathways related to AAA-related scores in GSE98278 (Figure 8B). Double-strand break repair *via* homologous recombination, protein phosphorylation, interferon-gamma-mediated signaling pathway, immune response, cellular response to DNA damage stimulus, T cell costimulation, T cell activation, innate immune response, and T cell receptor signaling pathway were positively enriched. In sum, AAA-related scores were associated with an immune hot microenvironment.

### Epigenetic characteristics of AAA-related score

**FIGURE 9:**
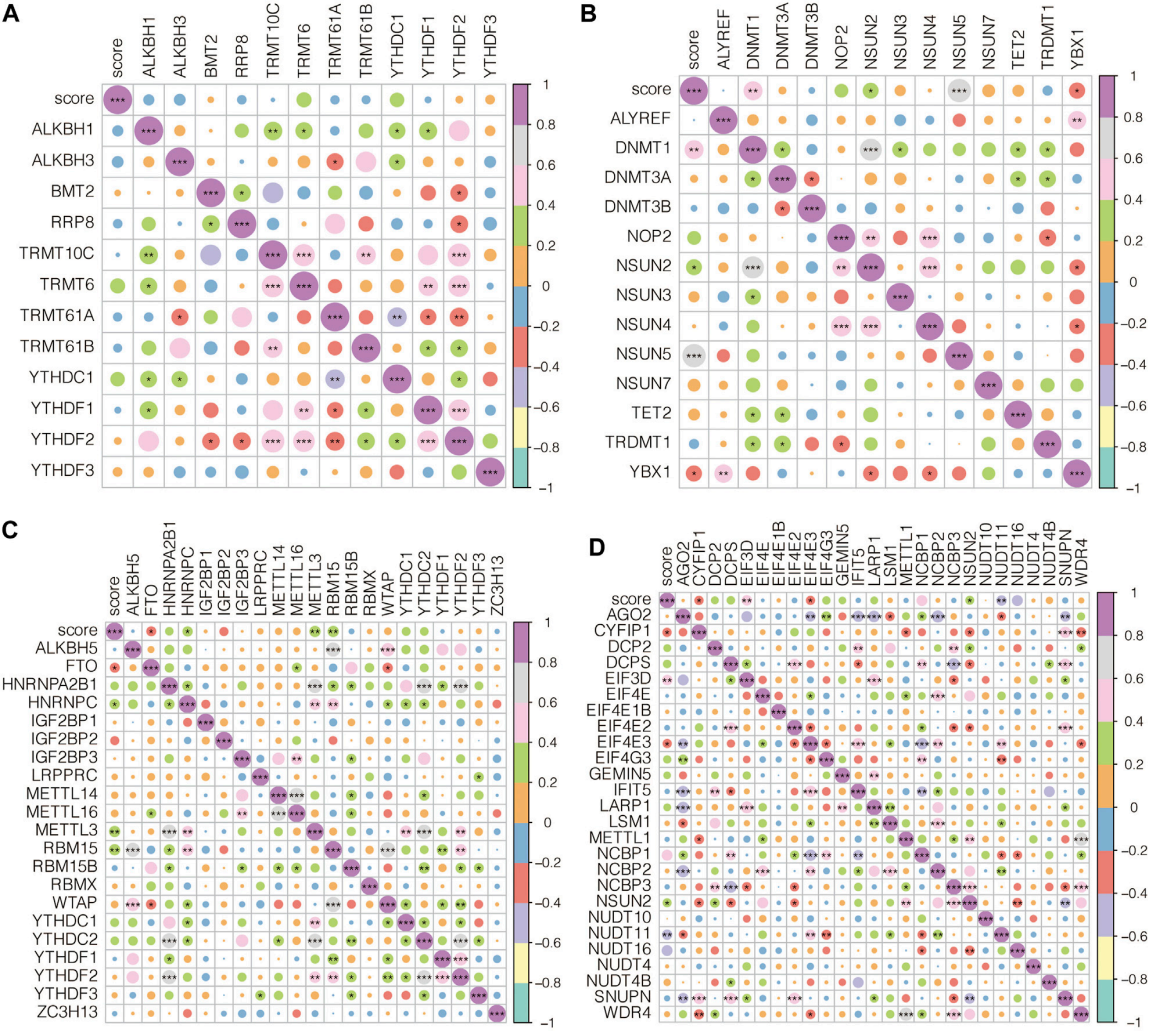
Epigenetic characteristics of AAA-related score. **(A)** The association between AAA-related score and m1A-related genes in GSE98278. **(B)** The association between AAA-related score and m5C-related genes in GSE98278. **(C)** The association between AAA-related score and m6A-related genes in GSE98278. **(D)** The association between AAA-related score and m7G-related genes in GSE98278.

The association between AAA-related score and m1A-related genes in GSE98278 is shown in Figure 9A, in which the association was insignificant. The association between AAA-related score and m5C-related genes in GSE98278 is shown in Figure 9B, in which AAA-related score was significantly associated with DNMT1, NSUN2, NSUN5, and YBX1. The association between AAA-related score and m6A-related genes in GSE98278 is shown in Figure 9C, in which AAA-related score was significantly associated with FTO, HNRNPC, METTL3, and RBM15. The association between AAA-related score and m7G-related genes in GSE98278 is shown in Figure 9D, in which AAA-related score was significantly associated with CYFIP1, EIF3D, EIF4E3, NSUN2, and NUDT11. AAA-related scores positively correlated with most m1A/m5C/m6A/m7G-related genes.

## Discussion

In the era of big data, excavating novel biomarkers or models in predicting the pathogenic mechanisms of AAA using large-scale bioinformatics analysis has been increasingly attractive. A previous study revealed differential expression of transcripts in the peripheral blood of AAA, indicating functional roles in proteolysis, inflammation, and apoptotic processes based on microarray-based gene expression profiling (Butt et al., 2016). A miRNA signature in AAA suggested that miRNAs play a role in AAA pathogenesis (Pahl et al., 2012). Besides, a lncRNA signature in AAA demonstrated that lncRNA candidates are related to the pathogenesis of AAA (Yang et al., 2016). The characteristic of inflammatory infiltration in the perivascular adipose tissue (PAT) surrounding AAA may be represented by a cell network dominated by FOS made up of activated mast cells, plasma cells, and Tfh cells (Ding et al., 2022). However, these models were not validated by *in vitro* validation. Besides, most of the model applications and universality were not so good.

In our study, WGCNA, as a generally used algorithm for determining feature-related genes, was used to determine the AAA-related genes for a comprehensive exploration of the pathogenic characteristics of AAA. Ubiquitin-mediated proteolysis, phosphoric ester hydrolase activity, nuclear ubiquitin ligase complex, and interleukin-1 beta secretion were highly enriched based on the WGCNA-derived module genes. Notably, a previous study has proven that potential therapeutic usefulness exists in altering how the ubiquitin-proteasome system function in vascular disorders (Wang et al., 2020). So, the AAA-related genes by WGCNA were believed to be reliable.

G2/M transition of mitotic cell cycle and inflammatory response were positively enriched based on limma-based DEGs between AAA and normal samples. In contrast, regulation of autophagy, RNA metabolic process, histone methylation, glycosaminoglycan metabolic process, histone H2A monoubiquitination, interleukin-27-mediated signaling pathway, and regulation of response to DNA damage stimulus were negatively enriched based on limma-based DEGs between AAA and normal samples. It is thought that one of the primary molecular pathways underpinning AAA development is inflammation in the AAA wall (Stepien et al., 2022). Therefore, the limma-based DEGs were also believed to be reliable.

By intersecting WGCNA-derived module genes and limma-based DEGs, the intersected genes had a solid potential to represent AAA. In this study, based on the WGCNA-derived module genes and limma-based DEGs between AAA and normal samples, the AAA-related score was developed using the random forest and LASSO machine learning algorithms. Artificial neural networks are a machine learning method that deep learning, a branch of artificial intelligence, employs to mine patterns and forecast outcomes from massive data sets. Research into the utility of deep learning in understanding the complicated biology of the disease has been boosted by the growing usage of deep learning across healthcare domains and the availability of extensively defined disease datasets (Tran et al., 2021). Although preliminary findings seem encouraging, this is a rapidly developing field with fresh insights into disease biology and deep learning. As machine learning has been widely proven with robust ability in model development, the AAA-related disease model was thought to be reliable.

Among the four model genes, UBE2K, TMEM230, VAMP7, and PUM2 were widely reported in human diseases. UBE2K could promote the progression of hepatocellular carcinoma *via* the regulation of c-Myc (Lei et al., 2022). TMEM230 was revealed to be a marker in Parkinson’s disease (Wang et al., 2021). VAMP7-dependent secretion of RTN3 was proven to regulate neurite growth (Wojnacki et al., 2020). PUM2 was a hazardous marker for mitochondrial dynamics and mitophagy during aging (D'Amico et al., 2019). Our study showed that UBE2K had significantly higher expression in AAA than in normal samples. In contrast, TMEM230, VAMP7, and PUM2 had significantly lower expression in AAA compared with normal samples. In other words, UBE2K was a hazardous marker in AAA, while TMEM230, VAMP7, and PUM were favorable markers in AAA. The clinical meaning and predictive value of UBE2K, TMEM230, VAMP7, and PUM2 were promising.

AAA-related score positively correlated with regulatory T cells (Tregs), plasma cells, activated dendritic cells, naïve B cells, activated CD4 memory T cells, and CD8 T cells. Double-strand break repair *via* homologous recombination, protein phosphorylation, interferon-gamma-mediated signaling pathway, immune response, cellular response to DNA damage stimulus, T cell costimulation, T cell activation, innate immune response, and T cell receptor signaling pathway were positively enriched in high AAA-related score AAA samples. Studies have shown the presence of multiple inflammatory cell types in AAA, such as macrophages, CD4^+^ T cells, and B cells, which play an essential role in the diseased aortic wall through phenotypic regulation (Okrzeja et al., 2022). In addition, recent evidence suggests that toll-like receptors, chemokine receptors, and complements in the innate immune system are involved in the progression of AAA (Li et al., 2018).

In our study, AAA-related scores positively correlated with most m1A/m5C/m6A/m7G-related genes. The AAA-related score was significantly associated with m5C-related genes DNMT1, NSUN2, NSUN5, and YBX1. The AAA-related score was significantly associated with m6A-related genes FTO, HNRNPC, METTL3, and RBM15. The AAA-related score was significantly associated with m7G-related genes CYFIP1, EIF3D, EIF4E3, NSUN2, and NUDT11. M1A, m5C, m6A, and m7G are new types of RNA methylation. M1A, m5C, m6A, and m7G are widely involved in regulating cardiovascular diseases, including heart failure, cardiac hypertrophy, aneurysm, and vascular calcification. Studies have also shown the expression pattern and functional significance of m6A-related genes in AAA (Zhang et al., 2021). m6A-related genes were found to play non-negligible roles in the occurrence of AAA ruptured (rAAA) (Fu et al., 2022). Besides, increased methylation levels of YTHDF3, FTO, and METTL14 were revealed to be associated with the progression of AAA (He et al., 2019b). METTL3 modulates m6A-dependent primary miR34a processing to induce AAA development and progression (Zhong et al., 2020). M6A could influence the circRNA-miRNA-mRNA network in hypoxia-mediated pulmonary hypertension. Moreover, the METTL3/YTHDF2/PTEN axis was proven to promote hypoxia-induced pulmonary artery hypertension (Zhou et al., 2021). YTHDF1 was reported to regulate pulmonary hypertension through the control of MAGED1 (Hu et al., 2021). NSUN2 influences autotaxin expression and T cell recruitment to control aneurysm development (Miao et al., 2021). In AAA, RBM15 knockdown decreased CASP3 expression in an m6A-dependent way (Fu et al., 2022). In AAA, YBX1 was discovered to be a direct target of GAS5, and it also collaborated with GAS5 to control the downstream target p21 through a positive feedback loop (He et al., 2019a). However, the specific mechanisms of other m1A/m5C/m6A/m7G epigenetic regulation (DNMT1, NSUN5, FTO, HNRNPC, CYFIP1, EIF3D, EIF4E3, NUDT11) in AAA have not been fully explored.

Our study developed a reliable AAA-related disease model for predicting immunity and m1A/m5C/m6A/m7G epigenetic regulation. The pathogenic roles of four model genes, UBE2K (hazardous), TMEM230 (favorable), VAMP7 (favorable), and PUM2 (favorable), in AAA, need further validation by *in vitro* and *in vivo* experiments.

## Data Availability

The original contributions presented in the study are included in the article/supplementary material, further inquiries can be directed to the corresponding author.

## References

[B1] Butt H. Z. Sylvius N. Salem M. K. Wild J. B. Dattani N. Sayers R. D. (2016). Microarray-based gene expression profiling of abdominal aortic aneurysm. Eur. J. Vasc. Endovasc. Surg. 52 (1), 47–55. 10.1016/j.ejvs.2016.03.016 27157464

[B2] Chaikof E. L. Dalman R. L. Eskandari M. K. Jackson B. M. Lee W. A. Mansour M. A. (2018). The Society for Vascular Surgery practice guidelines on the care of patients with an abdominal aortic aneurysm. J. Vasc. Surg. 67 (1), 2–77.e2. 10.1016/j.jvs.2017.10.044 29268916

[B3] D'Amico D. Mottis A. Potenza F. Sorrentino V. Li H. Romani M. (2019). The RNA-binding protein PUM2 impairs mitochondrial dynamics and mitophagy during aging. Mol. Cell 73 (4), 775–787.e10. 10.1016/j.molcel.2018.11.034 30642763 PMC6396316

[B4] Ding S. Gan T. Xiang Y. Zhu X. Jin Y. Ning H. (2022). FOS gene associated immune infiltration signature in perivascular adipose tissues of abdominal aortic aneurysm. Gene 831, 146576. 10.1016/j.gene.2022.146576 35568340

[B5] Fu C. Feng L. Zhang J. Sun D. (2022). Bioinformatic analyses of the role of m6A RNA methylation regulators in abdominal aortic aneurysm. Ann. Transl. Med. 10 (10), 547. 10.21037/atm-22-1891 35722410 PMC9201186

[B6] He X. Wang S. Li M. Zhong L. Zheng H. Sun Y. (2019a). Long noncoding RNA GAS5 induces abdominal aortic aneurysm formation by promoting smooth muscle apoptosis. Theranostics 9 (19), 5558–5576. 10.7150/thno.34463 31534503 PMC6735383

[B7] He Y. Xing J. Wang S. Xin S. Han Y. Zhang J. (2019b). Increased m6A methylation level is associated with the progression of human abdominal aortic aneurysm. Ann. Transl. Med. 7 (24), 797. 10.21037/atm.2019.12.65 32042813 PMC6989874

[B8] Hu L. Wang J. Huang H. Yu Y. Ding J. Yu Y. (2021). YTHDF1 regulates pulmonary hypertension through translational control of MAGED1. Am. J. Respir. Crit. Care Med. 203 (9), 1158–1172. 10.1164/rccm.202009-3419OC 33465322

[B9] Lei X. Hu X. Lu Q. Yao Y. Sun W. Ma Q. (2022). UBE2K promotes the malignant progression of hepatocellular carcinoma by regulating c-Myc. Biochem. Biophys. Res. Commun. 638, 210–218. 10.1016/j.bbrc.2022.11.046 36481361

[B10] Li D. Li K. Zhang W. Yang K. W. Mu D. A. Jiang G. J. (2022). The m6A/m5C/m1A regulated gene signature predicts the prognosis and correlates with the immune status of hepatocellular carcinoma. Front. Immunol. 13, 918140. 10.3389/fimmu.2022.918140 35833147 PMC9272990

[B11] Li H. Bai S. Ao Q. Wang X. Tian X. Li X. (2018). Modulation of immune-inflammatory responses in abdominal aortic aneurysm: Emerging molecular targets. J. Immunol. Res. 2018, 7213760. 10.1155/2018/7213760 29967801 PMC6008668

[B12] Marquez-Sanchez A. C. Koltsova E. K. (2022). Immune and inflammatory mechanisms of abdominal aortic aneurysm. Front. Immunol. 13, 989933. 10.3389/fimmu.2022.989933 36275758 PMC9583679

[B13] Miao Y. Zhao Y. Han L. Ma X. Deng J. Yang J. (2021). NSun2 regulates aneurysm formation by promoting autotaxin expression and T cell recruitment. Cell Mol. Life Sci. 78 (4), 1709–1727. 10.1007/s00018-020-03607-7 32734582 PMC11073013

[B14] Okrzeja J. Karwowska A. Blachnio-Zabielska A. (2022). The role of obesity, inflammation and sphingolipids in the development of an abdominal aortic aneurysm. Nutrients 14 (12), 2438. 10.3390/nu14122438 35745168 PMC9229568

[B15] Pahl M. C. Derr K. Gabel G. Hinterseher I. Elmore J. R. Schworer C. M. (2012). MicroRNA expression signature in human abdominal aortic aneurysms. BMC Med. Genomics 5, 25. 10.1186/1755-8794-5-25 22704053 PMC3507654

[B16] Sakalihasan N. Limet R. Defawe O. D. (2005). Abdominal aortic aneurysm. Lancet 365 (9470), 1577–1589. 10.1016/S0140-6736(05)66459-8 15866312

[B17] Shao D. Li Y. Wu J. Zhang B. Xie S. Zheng X. (2022). An m6A/m5C/m1A/m7G-Related long non-coding RNA signature to predict prognosis and immune features of glioma. Front. Genet. 13, 903117. 10.3389/fgene.2022.903117 35692827 PMC9178125

[B18] Stepien K. L. Bajdak-Rusinek K. Fus-Kujawa A. Kuczmik W. Gawron K. (2022). Role of extracellular matrix and inflammation in abdominal aortic aneurysm. Int. J. Mol. Sci. 23 (19), 11078. 10.3390/ijms231911078 36232377 PMC9569530

[B19] Tran K. A. Kondrashova O. Bradley A. Williams E. D. Pearson J. V. Waddell N. (2021). Deep learning in cancer diagnosis, prognosis and treatment selection. Genome Med. 13 (1), 152. 10.1186/s13073-021-00968-x 34579788 PMC8477474

[B20] Wang B. Cai W. Ai D. Zhang X. Yao L. (2020). The role of deubiquitinases in vascular diseases. J. Cardiovasc Transl. Res. 13 (2), 131–141. 10.1007/s12265-019-09909-x 31823221

[B21] Wang X. Whelan E. Liu Z. Liu C. F. Smith W. W. (2021). Controversy of TMEM230 associated with Parkinson's disease. Neuroscience 453, 280–286. 10.1016/j.neuroscience.2020.11.004 33212219

[B22] Wanhainen A. Verzini F. Van Herzeele I. Allaire E. Bown M. Cohnert T. (2019). Editor's choice - European society for vascular surgery (ESVS) 2019 clinical practice guidelines on the management of abdominal aorto-iliac artery aneurysms. Eur. J. Vasc. Endovasc. Surg. 57 (1), 8–93. 10.1016/j.ejvs.2018.09.020 30528142

[B23] Wojnacki J. Nola S. Bun P. Cholley B. Filippini F. Presse M. T. (2020). Role of VAMP7-dependent secretion of reticulon 3 in neurite growth. Cell Rep. 33 (12), 108536. 10.1016/j.celrep.2020.108536 33357422

[B24] Wu Y. Zhan S. Xu Y. Gao X. (2021). RNA modifications in cardiovascular diseases, the potential therapeutic targets. Life Sci. 278, 119565. 10.1016/j.lfs.2021.119565 33965380

[B25] Yang Y. G. Li M. X. Kou L. Zhou Y. Qin Y. W. Liu X. J. (2016). Long noncoding RNA expression signatures of abdominal aortic aneurysm revealed by microarray. Biomed. Environ. Sci. 29 (10), 713–723. 10.3967/bes2016.096 27927271

[B26] Zhang L. Yao L. Zhou W. Tian J. Ruan B. Lu Z. (2021). miR-497 defect contributes to gastric cancer tumorigenesis and progression via regulating CDC42/ITGB1/FAK/PXN/AKT signaling. Mol. Ther. Nucleic Acids 25, 567–577. 10.1016/j.omtn.2021.07.025 34589278 PMC8463315

[B27] Zhong L. He X. Song H. Sun Y. Chen G. Si X. (2020). METTL3 induces AAA development and progression by modulating N6-methyladenosine-dependent primary miR34a processing. Mol. Ther. Nucleic Acids 21, 394–411. 10.1016/j.omtn.2020.06.005 32650237 PMC7347714

[B28] Zhou W. Wang C. Chang J. Huang Y. Xue Q. Miao C. (2021). RNA methylations in cardiovascular diseases, molecular structure, biological functions and regulatory roles in cardiovascular diseases. Front. Pharmacol. 12, 722728. 10.3389/fphar.2021.722728 34489709 PMC8417252

